# Facile synthesis of copper, nickel and their bimetallic nanoparticles: optical and structural characterization

**DOI:** 10.1186/s11671-025-04197-8

**Published:** 2025-02-11

**Authors:** Abdul Waheed Aman, Ganesan Krishnan, Mohammad Abdullah Sadiqi, Mahmood Alhajj, Nurul Hidayat

**Affiliations:** 1 Department of Physics, Faculty of Education, Helmand University, Lashkar Gah, 3901 Helmand Afghanistan; 2 Department of Physics and Laser Center, Faculty of Science, Universiti Teknologi Malaysia, Skudai, 81310 Johor Malaysia; 3 Department of Physics, Faculty of Mathematics and Natural Sciences, Universitas Negeri Malang, Jl. Semarang 5, Malang, 65145 Indonesia; 4 Center of Science and Engineering, Universitas Negeri Malang, Jl. Semarang 5, Malang, 65145 Indonesia

**Keywords:** Pulsed laser ablation, Cu–Ni nanoparticles, Plasmonic nanoparticles, Band gap

## Abstract

This paper reports the synthesis and characterization of some spherical crystalline copper (Cu), nickel (Ni), and their bimetallic (Cu–Ni) nanoparticles (NPs) produced in deionized (DI) water via pulse laser ablation in liquid (PLAL) technique. XRD and SAED patterns showed the high crystallinity of the synthesized nanostructures with face-centered cubic structure. The TEM and HRTEM images revealed the nucleation of spherical nanocrystals with a size range of 2.5**–**25 nm, and no remarkable aggregation was seen. Some core–shell oxidized Cu and Ni nanostructures related to the dissolved oxygen in DI water were detected. The UV–Vis absorption spectra at different aging times showed that pure metal nanoparticles are more stable than bimetallic nanoparticles. Localized surface plasmon resonance (LSPR) absorption peaks were recorded at 645 nm and 596 nm wavelengths for Cu and Cu–Ni colloidal solutions, respectively. The PL emission peaks at shorter wavelengths indicated that synthesized nanoparticles are blue luminescent. The achievement of pure and spherical copper, nickel, and their bimetallic nanoparticles with enhanced optical properties could be beneficial for advancing photocatalysis and antibacterial activity.

**Supplementary Information:**

The online version contains supplementary material available at 10.1186/s11671-025-04197-8.

## Introduction

In recent years, the synthesis and applications of metal nanoparticles (MNPs) have attracted considerable attention due to their particular optical, magnetic, catalytic, electronic, and chemical properties compared to their bulk counterparts [1–3]. Due to their novel properties, MNPs have various practical applications in bioengineering [4], antibacterial activity [5–7], anticancer activity [8], solar cells [9, 10], drug delivery [11], and dye decomposition catalysis [12]. Among MNPs, copper nanoparticles (Cu NPs) with enhanced and tunable localized surface plasmon resonance (LSPR) band in the red region of the absorption spectrum are used in various plasmonic applications [13]. On the other hand, nickel nanoparticles (Ni NPs) with distinct magnetic and optical properties are widely used as gas and temperature sensors and optical filters [14].

Recent studies have shown that plasmonic metal nanocrystals have the potential to photocatalyze different chemical reactions. Thus, numerous efforts have been made to synthesize novel photocatalyst nanostructures with tunable catalytic properties under visible and UV light exposure. In this regard, plasmonic bimetallic NPs developed as a new class of photocatalysts as they demonstrate exceptional physiochemical properties lacking in their constituents or bulk counterparts. Recently, Cu–Ni bimetallic NPs have become of great interest due to their catalytic and antibacterial activities. They are widely used as catalysts in various chemical reactions such as the reduction of 4-nitrophenol [15], ethanol steam reforming [16], photoreduction of volatile organic compounds [17], and partial oxidation of methanol [18]. In addition, the intense antibacterial effects of Cu–Ni NPs against standard human pathogens have also been reported in Ref. [3, 19]. Meanwhile, Cu–Ni NPs are also well known for their magnetic properties, making them applicable in biomedical applications, such as magnetic-mediated hyperthermia [20].

A variety of physical and chemical methods are available for the synthesis of MNPs such as magnetron sputtering [21], metal-vapor synthesis [22], chemical reduction [23], microemulsion [24], sonochemical [25], and sol–gel [26]. However, these methods need long reaction times, chemical reagents, and high bulk temperatures. In a sense, it still remains a challenge to synthesize pure bimetallic NPs in a facile and controlled way. It would be of great importance to develop a facile synthetic strategy for the fabrication of monometallic and bimetallic NPs due to the increasing demand for these NPs in various fields of application. In contrast to other synthetic methods, pulsed laser ablation in liquid (PLAL) is a suitable and versatile method for synthesizing MNPs with high purity and crystallinity. Since the final product is free from hazardous or toxic byproducts, the NPs synthesized by this method are strongly desirable in biological applications. The structure, composition, size, and morphology of NPs synthesized by PLAL method, depend upon the laser parameters such as laser wavelength, pulse length, repetition rate, and the liquid environment [27–29].

In the laser ablation method, a high-power laser pulse interacts with the surface of the metal target. Thus the energy transferred via the pulsed laser beam increases the temperature of the irradiated surface to the melting point. After further energy deposition, the phase of target material changes from liquid to vapor state and then to plasma. This is followed by a shockwave emission, the ejection and expansion of a mixture of atoms, atomic clusters, and nanoparticles is known as plasma plume. Condensation of the vapor-phase atoms and clusters leads to the further formation and growth of NPs [30, 31].

During PLAL, the ejected molten material interacts with the solvent, providing a reactive environment for metal oxidation. Oxidation of NPs takes place in two phases. An earliest and superficial oxidation occurs first, wherein the ablated superheated droplets surrounded by water molecules result in the oxidation of NPs. The second step is a later and slower oxidation, which is sensitive to the repetition rate of the laser and is completed only well after the end of ablation. At a lower repetition rate (a longer delay between successive pulses), this oxidation proceeds together with the rate of MNPs production. This could be due to the longer ablation time leading to the longer interaction of NPs with water. However, the oxidation of MNPs after the end of PLAL is independent of the repetition rate [14, 32].

In present work, we prepared Cu NPs, Ni NPs, and Cu–Ni bimetallic NPs directly via laser ablation of corresponding target plates in deionized (DI) water. This fast, simple, and green technique could avoid multistep chemical processing, long reaction times, and high bulk temperatures, which is advantageous for various applications. The morphology and optical properties of synthesized MNPs were systematically characterized using several analytical techniques. Meanwhile, the stability of samples was investigated by determining the optical absorbance as a function of aging time. The obtained findings suggest that Cu–Ni NPs could be promising candidates for photocatalytic and antibacterial activities. Meanwhile, only limited efforts have been made to synthesize the Cu–Ni bimetallic NPs directly from the laser ablation of their alloy target. Ali et al. [33] synthesized Cu–Ni bimetallic NPs from a bulk alloy target with higher antibacterial activities. To the authors’ knowledge, the optical characteristics of Cu–Ni NPs have not been well studied in the literature.

## Experimental setup

**Fig. 1:**
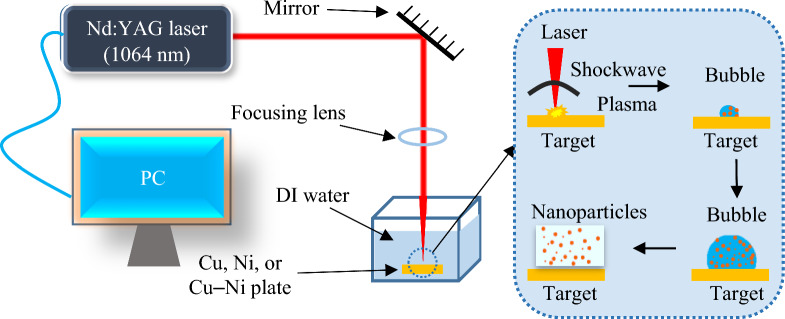
Schematic illustration of PLAL experimental setup and nanoparticles growth mechanism

Figure 1 displays the schematic representation of PLAL experiment. In this typical experimental setup, commonly known as the batch method, a focused laser beam is directed on the surface of target in a liquid [34]. In this work, a nanosecond Nd:YAG pulsed laser with a beam diameter of 1.5 mm was used as an irradiation source of ablation. The laser operated at a repetition rate of 10 Hz, wavelength of 1064 nm, and a pulse width of 8 ns. The output pulse energy was calibrated using a Newport power meter set to 160 mJ for all samples. A plane mirror adjusted at the optical path was employed to direct the laser beam toward the target. The laser beam was focused onto the target using a concave lens with a focal length of 100 mm. High-purity Cu and Ni plates (over 99.99%) and Cu–Ni (60% Cu and 40% Ni) alloy target with 1 × 1 cm^2^ surface area and 2 mm thickness immersed in DI water were ablated for 8 min. First, the target plates were ground with 600 and 700 grit-sized sandpaper to remove the dirt and other contaminants, the targets were washed with acetone and followed by an ultrasonic bath for 15 min in DI water before the PLAL process. Next, the cleaned plates were placed at the bottom of a quartz cuvette filled with 6 ml of DI water. The cuvette was slowly rotated at a rotational stage to disperse the cloud of NPs in the solution evenly and to avoid the formation of craters on the target surface.

Several experiments were conducted at room temperature to characterize the prepared samples. A Rigaku SmartLab XRD diffractometer (Cu-${K}_{\alpha 1}$ radiations, λ = 1.5406 Å) was employed to study the crystalline structures phases of grown NPs. XRD patterns were recorded from 20° to 90° of $2\theta$ after a few drops of each colloidal solution were dried on a Si wafer. The shape, size distribution, and selected area electron diffraction (SAED) pattern were examined through a high-resolution transmission electron microscope (JEM-ARM 200F, HR-TEM 200kV). A drop from each colloidal solution was placed on a carbon-coated copper grid and dried inside a vacuum chamber at room temperature. The size distribution histograms of produced NPs were obtained by statistical evaluation of nearly 100 NPs diameter of each sample from TEM micrographs. A UV–Vis spectrophotometer (Shimadzu UV—3600 Plus) was used to record the optical absorption spectra ranging from 250 to 800 nm. The PL spectra were measured in the visible region using a Perkin-Elmer Luminescence LS55 spectrometer with a 350 nm excitation. The existence of vibrational bonds in the prepared samples were determined using an ATR-IR spectrometer (Perkin-Elmer Frontier™) in the range of 500–4000 cm^−1^.

## Results and discussion

### Morphology and structure

**Fig. 2:**
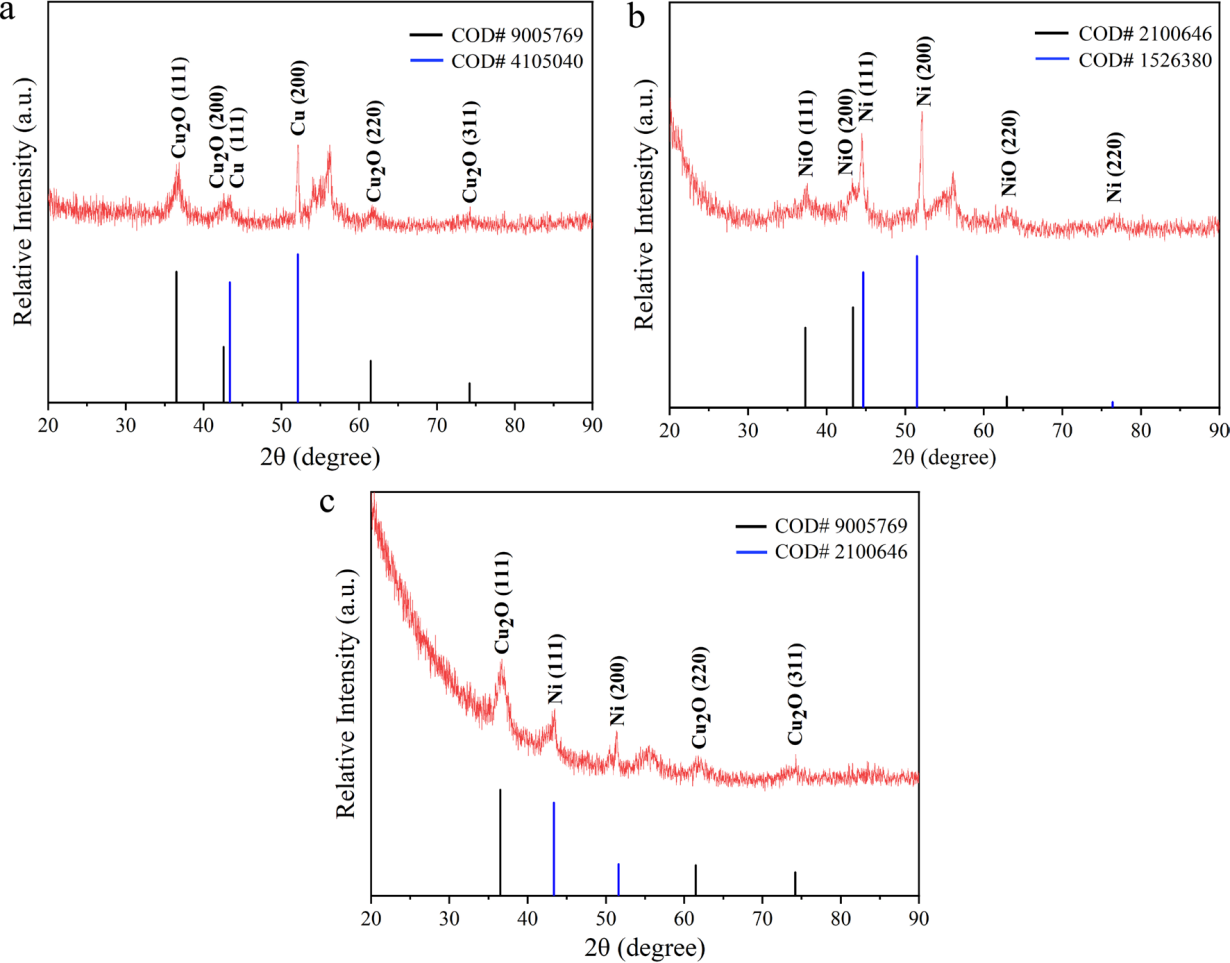
XRD patterns of prepared MNPs with corresponding standard data plotted as vertical lines: **a** Cu NPs, **b** Ni NPs, and **c** Cu–Ni NPs

The XRD patterns with corresponding standard data of as-grown nanocrystals dried on Si substrate are shown in Fig. 2. The observed diffraction peaks for each sample match well with the corresponding standard patterns and confirm the polycrystalline structure of synthesized NPs. An additional peak detected at 56° was attributed to the (311) lattice orientation of the Si substrate. No further diffraction peaks correlated to other impurity crystalline phases were recorded. XRD data clearly displayed the oxidation of obtained NPs, which was attributed to the reactivity of Cu and Ni with the oxygen dissolved in water.

In Fig. 2a, characteristic peaks at 43.32° and 50.76° of 2 $\theta$ values are ascribed to the (111) and (200) lattice directions of copper with face-centered cubic (fcc) structure. Meanwhile, the occurrence of diffraction peaks at 36.48°, 42.61°, 61.87°, and 74.15° is related to the (111), (200), (220), and (311) planes of the cuprite (Cu_2_O) structure, respectively. The 44.48°, 52.13°, and 76.44° peaks, in Fig. 2b, can be indexed as the (111), (200), and (220) planes of Ni fcc structure, while the three more peaks at 37.35°, 43.26°, and 62.91° are attributed to the (111), (200), and (220) lattice orientations of nickel oxide (NiO) phase. Similar diffraction peaks for Ni NPs synthesized in DI water were observed at 2 $\theta$ of 44.35, 51.8, and 76.3 assigned to the (111), (200), and (220) planes of fcc Ni and 37.2, 43.2, and 62.85 belong to the (111), (200), and (220) planes of NiO in Ref. [35]. They also showed that the structure of Ni NPs prepared via PLAL strongly depends on the solvents used in the ablation processes. Argueta-Figueroa et al*.* [19] reported the fabrication of Cu NPs and Ni NPs with the same structures.

Figure 2c shows the XRD patterns of Cu–Ni NPs and proves the presence of both copper and nickel phases. The diffraction peaks at 36.38°, 61.33°, and 74.14° are assigned as the (111), (220), and (311) planes of the cuprite structure. Besides, the 43.98° and 51.76° peaks are related to the (111) and (200) crystal planes of fcc nickel. Similar diffraction peaks such as 44. 6°, 51.8°, and 74.14° belong to (111), (200), and (220) of Cu–Ni bimetallic NPs were reported in Ref. [36]. The XRD data imply that Cu–Ni NPs are successfully synthesized from bulk alloy target. The average crystallite size of each sample was calculated using Scherrer equation (shown in Table 1).

**Table 1:** The average particle size, crystallite size, energy band gap, and SPR and PL peak positions of PLAL-asserted prepared NPs, compared to other findings reported in the literature

Samples	Synthesis Method	Average particle size in diameter (nm)	Crystallite size (nm)	SPR peak position (nm)	${E}_{g}$ from Tauc plot (eV)	PL peak position (nm)	Refs.
Cu NPs	PLAL	7.2 ± 1.4	6.1	645	2.93	436	Present work
Ni NPs	PLAL	10.2 ± 2.4	7.4	**–**	3.14	397	Present work
Cu–Ni NPs	PLAL	9.5 ± 2.8	6.9	596	3.05	406	Present work
Cu–Ni NPs	Sol**–**gel	15–20	17–19	–	–	–	[20]
Cu–Ni NPs	USP-HR	300, 510	40, 34	–	–	–	[54]
Cu–Ni NPs	EEW	21–56	–	–	–	–	[55]
Cu–Ni NPs	Thermal reduction	6.9–27.3	–	–	–	–	[15]

**Fig. 3:**
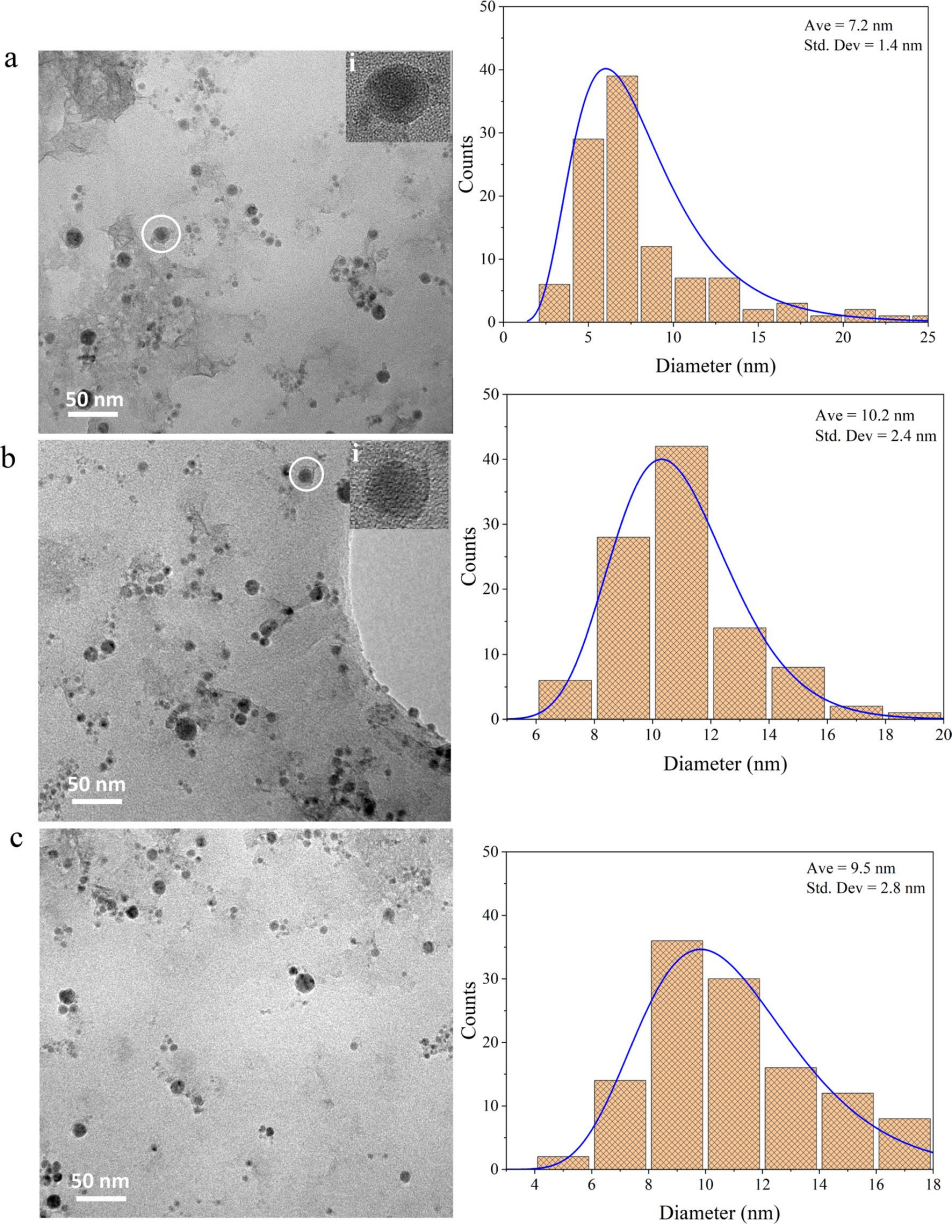
TEM images with corresponding size distribution histograms: **a** Cu NPs; **b** Ni NPs; **c** Cu–Ni NPs. Inset **i** exhibits magnified image of marked core–shell nanoparticlesl

The powerful TEM technique was done for further morphological and structural investigations of the produced nanocrystals. TEM results and corresponding size distribution histograms are represented in Fig. 3. The formation of spherical NPs with no significant aggregation and relatively narrow size distribution were confirmed. Insets i, in Fig. 3a and b, illustrate the enlarged scale of noticed core–shell nanostructures of Cu NPs and Ni NPs, respectively. The average particle size with standard deviation was estimated 7.2 ± 1.4 nm, 10.2 ± 2.4 nm, and 9.5 ± 2.8 nm for Cu NPs, Ni NPs, and Cu–Ni NPs, respectively.

Elemental composition of each sample was confirmed through the energy-dispersive X-ray (EDX) analysis. EDX examination was performed during TEM analysis, and the results are stated in the Supporting Information file (Fig. S1).

**Fig. 4:**
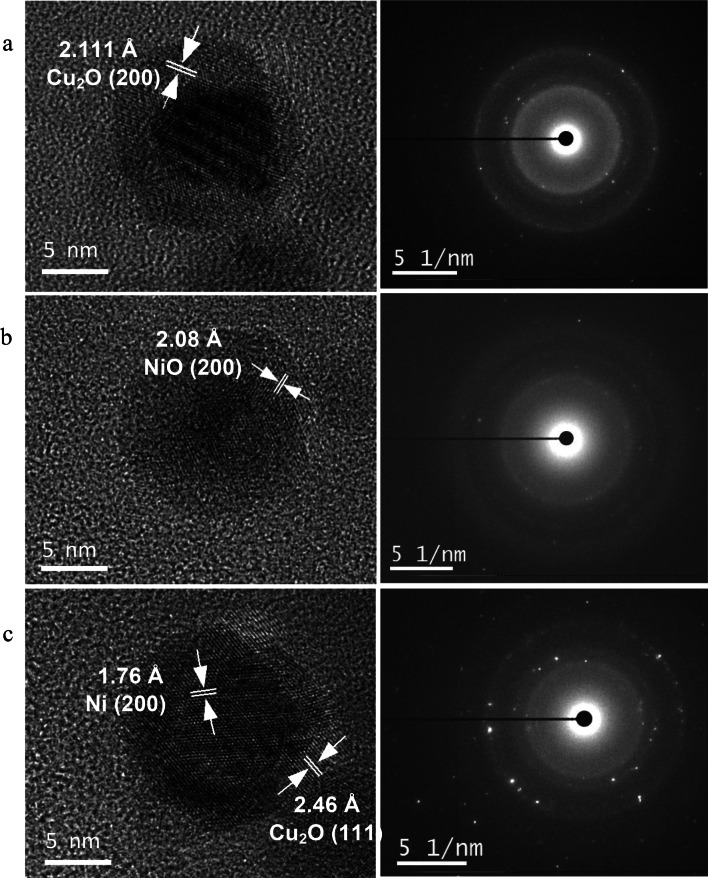
HR-TEM micrographs with corresponding SAED patterns: **a** Cu NPs; **b** Ni NPs; **c** Cu–Ni NPs

Figure 4 presents magnified HR-TEM images associated with SAED patterns of a single particle of each sample. A Cu2O (200) lattice fringe of a Cu NP with the 2.111 Å spacing is shown in Fig. 4a. A NiO (200) plane of a Ni NP with a lattice spacing of 2.08 Å was calculated (Fig. 4b). The HR-TEM micrographs also revealed core–shell structure of Cu NPs and Ni NPs which suggest a post-growth of oxide shell over the nanocrystalline core in oxygen-rich DI water. The lattice orientations of Ni (200) close to the center and Cu_2_O (111) at the edge of the particle were measured for Cu–Ni sample (Fig. 4c), which indicates the formation of Cu–Ni bimetallic NPs with a Ni-rich core and Cu-rich shell. These NPs with Cu at the surface are easily oxidized due to the higher oxidation potential of Cu.

In addition, SAED pattern (Fig. 4) corresponding to the selected area of each particle confirmed their crystallinity with growth orientation along various lattice planes with the detection of bright circular spots and concentric rings. These findings are consistent with the above-stated XRD data and approve the crystallinity of synthesized NPs.

### Optical characterization

**Fig. 5:**
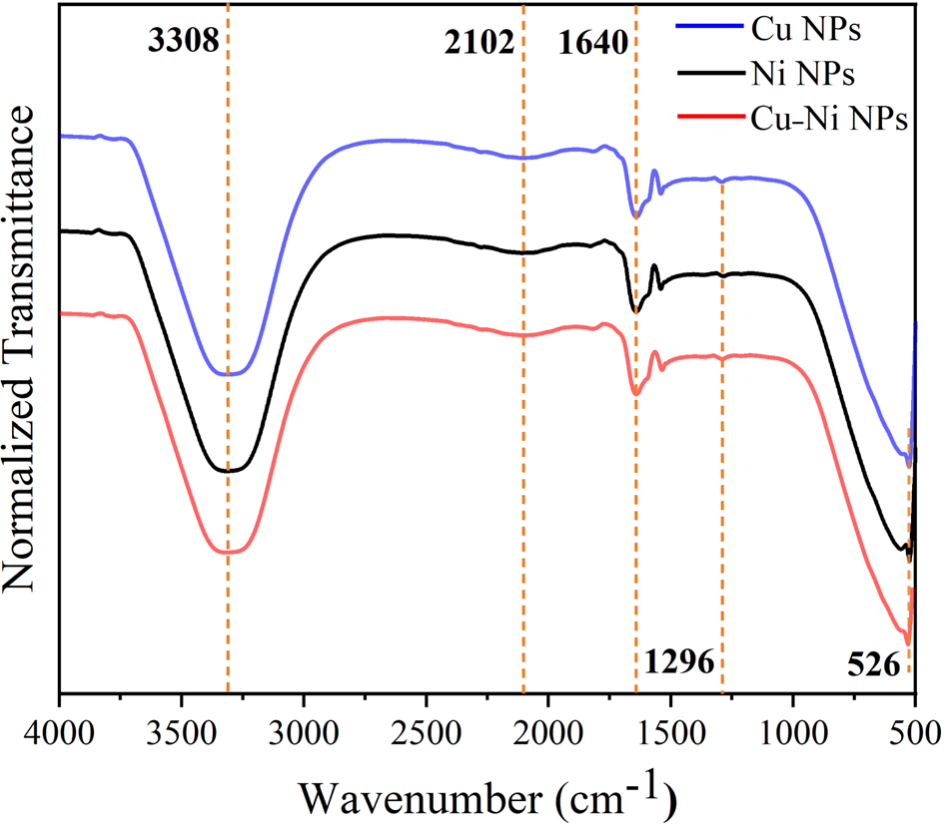
Room temperature ATR–IR spectra of synthesized MNPs

ATR–IR spectral analysis (in the range of 500–4000 cm^−1^) of the as-prepared NPs dispersed in DI water was performed at room temperature (Fig. 5). ATR–IR spectroscopy is a powerful analytical technique for investigating the molecular vibrational modes at the solid–liquid interface, wherein the IR radiation is restricted close to the surface of the catalyst to minimize the signal contribution of liquid to the recorded IR spectrum [37, 38]. The broad and enhanced IR absorption band at 3308 cm^−1^ was ascribed to the stretching oscillation of the hydroxyl (–OH) group of water attached to the surface of produced NPs [39, 40]. Furthermore, the appearance of a weaker absorption band at 2102 cm^−1^ was assigned to C=C-like stretching vibration [41]. The vibration at 1640 cm^−1^ was related to the C=O stretching of carbonyl group [42]. The tiny band at 1296 cm^−1^ was approved to the wagging and twisting vibrations while the strong IR absorption bands at lower wavenumbers were due to the rocking bending [42, 43] The existence of absorption bands around 526 cm^−1^ could be related to the oxygen bond on the surface of NPs because of the oxidation [3]. In brief, the detection of no absorption peaks related to contaminants in ATR–IR spectra confirmed the surface purity of the achieved MNPs, implying their usefulness for practical applications in biomedical, photocatalysis, and solar cells.

**Fig. 6:**
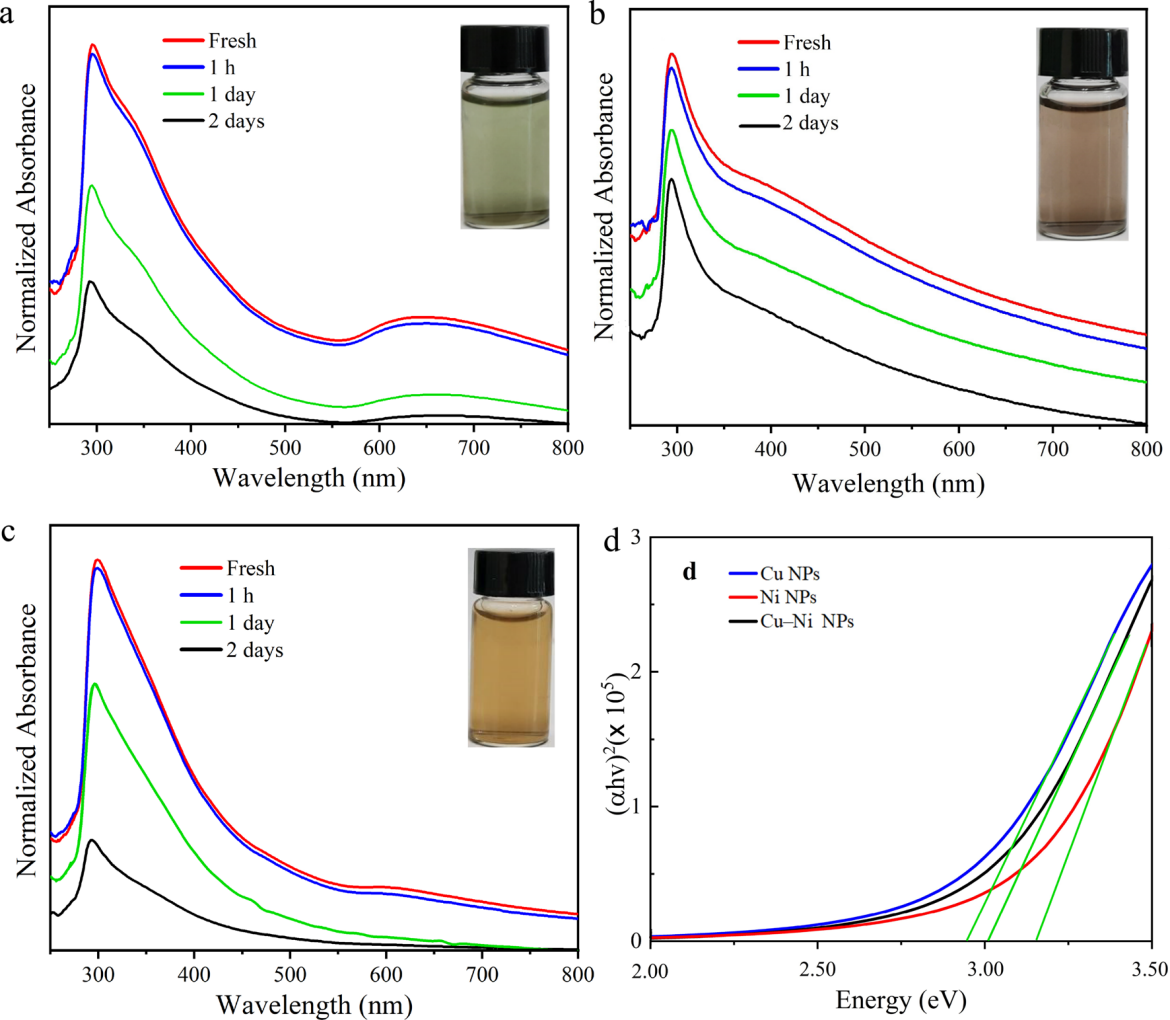
UV–Vis absorption spectra of prepared colloidal solutions: **a** Cu NPs; **b** Ni NPs; **c** Cu–Ni NPs. **d** Tauc plots

Figure 6 represents the UV–Vis absorption spectra of fresh samples as well as after one hour, one day, and two days of their storage time. After laser ablation process, the color of as-synthesized Cu, Ni, and Cu–Ni colloidal solutions changed from transparent (DI water) to emerald green, dark gray, and brown-yellow (bottles shown in Fig. 6), respectively. This confirmed the generation of well-dispersed NPs in DI water. The UV–Vis absorption analysis showed that copper and nickel colloidal solutions are relatively steady compare to bimetallic nanocoloids.

In the absorption spectra, the intense absorption at shorter wavelengths is attributed to the interband electronic transitions between the respective d and sp bands that are allowed transitions and defined by band structures. On the other hand, the absorption in visible or infrared regions is due to the intraband transitions within the sp band states, also called localized surface plasmon resonance (LSPR). LSPR occurs as collective oscillations of electrons in MNPs in response to electromagnetic radiation when its frequency matching with the intrinsic oscillator frequency of the NPs. The LSPR is highly enhanced in noble metal NPs but becomes weaker in other transition MNPs. The intensity and position of LSPR absorptions strongly depend on the nanocrystals size, shape, distribution, surrounding medium, and polarization of incident radiation. In contrast, interband transitions of each metal arise from an intrinsic absorption within a certain region of the spectrum and are not notably sensitive to geometric factors [44, 45]. The gradual reduction in absorption intensity associated with the storage time of each sample is correlated to the aggregation which causes an increment in particle size [46].

The UV–Vis absorption spectra of Cu colloidal solution are exhibited in Fig. 6a. The absorption peak related to LSPR of Cu NPs is observed at 645 nm, which is in good agreement with the literature [32]. Because of this wide band absorbance in the red region, the color of the colloidal solution looks green. The absorption spectra of Ni NPs colloid at different aging times are shown in Fig. 6b. A broad absorbance in the visible region results in the dark-brown color of the colloidal solution. A broad and less intense LSPR band is visible at 596 nm for fresh sample Cu–Ni nanocolloid which makes the colloidal solution look brown-yellow in color (Fig. 6c). Non-uniform particle size results in a broad absorbance spectrum due to quantum confinement, where smaller particles exhibit blue shifts, and larger ones show red shifts, leading to spectral broadening. Irregular shapes and aggregation also alter the surface plasmon resonance, further contributing to this broadening.

Rawat et al. [47] demonstrated that the fluence, solvent concentration, and ablation duration significantly influence the characteristics of Cu NPs. Notably, the study found that increasing the ablation time from 10 to 60 min led to a blue shift in the SPR peak from 590 to 585 nm, indicating particle size reduction. Additionally, variations in solvents (ethylene glycol, DI water, ethanol) resulted in distinct absorption spectra and band gaps, with CuO NPs exhibiting higher band gaps in ethylene glycol (3.64 eV) compared to DI water (2.97 eV) and ethanol (3.40 eV), emphasizing the critical role of solvent effects on NP formation. Meanwhile, changes in the liquid medium lead to the size tuning of Ni NPs and Cu NPs [48].

For comparison, the position, intensity, and full width at half maximum (FWHM) of LSPR peak are plotted as functions of aging time (Supporting Information file, Fig. S2). The disappearance of LSPR peak and faster decline in absorption intensity of Cu–Ni colloidal solution after one day from the end of PLAL is due to quicker aggregation of these NPs.

Furthermore, in contrast to their bulk components, monometallic and alloy NPs have optical band gaps that can be widened for improved photocatalytic activities. The band gap energy ($${E}_{g})$$ of the PLAL-grown NPs was measured from the linear part of the absorption edge of UV–Vis spectra using the Tauc method. The value of $${E}_{g}$$ was attained from [43, 49, 50]:

$$\alpha hv = C\left( {hv - E_{g} } \right)^{r}$$

in which $hv$, $C$, $\alpha$, and $r$ relate to the photon energy, a constant magnitude, absorption coefficient, and a power factor. The optical absorption is said to be direct or indirect allowed transition if r = 1/2 or 2, respectively. For direct allowed transition, r = 1/2 was taken into account. The optical band gap energy was calculated from the extrapolation of the linear portion of ${(\alpha hv)}^{2}$ versus $hv$ graph to the abscissa ${((\alpha hv)}^{2}=0)$. The estimated values of ${E}_{g}$ for Cu NPs, Ni NPs, and Cu–Ni NPs obtained from Tauc plots were 2.93 $\text{eV}$, 3.14 $\text{eV}$, and 3.05 $\text{eV}$, respectively. The optical band gap energy strongly depends on the size of NPs, wherein the stronger quantum size effect in smaller NPs leads to a wide gap in the electronic density of states. The presence of wider optical band gap was due to overlapping orbitals and less number of atoms in smaller NPs, responsible for the shrinkage of the energy band width. These results are consistent with the TEM observation as tinier NPs due to quantum confinement effect showed narrowing of the valance band (VB) and conduction band (CB) [51].

**Fig. 7:**
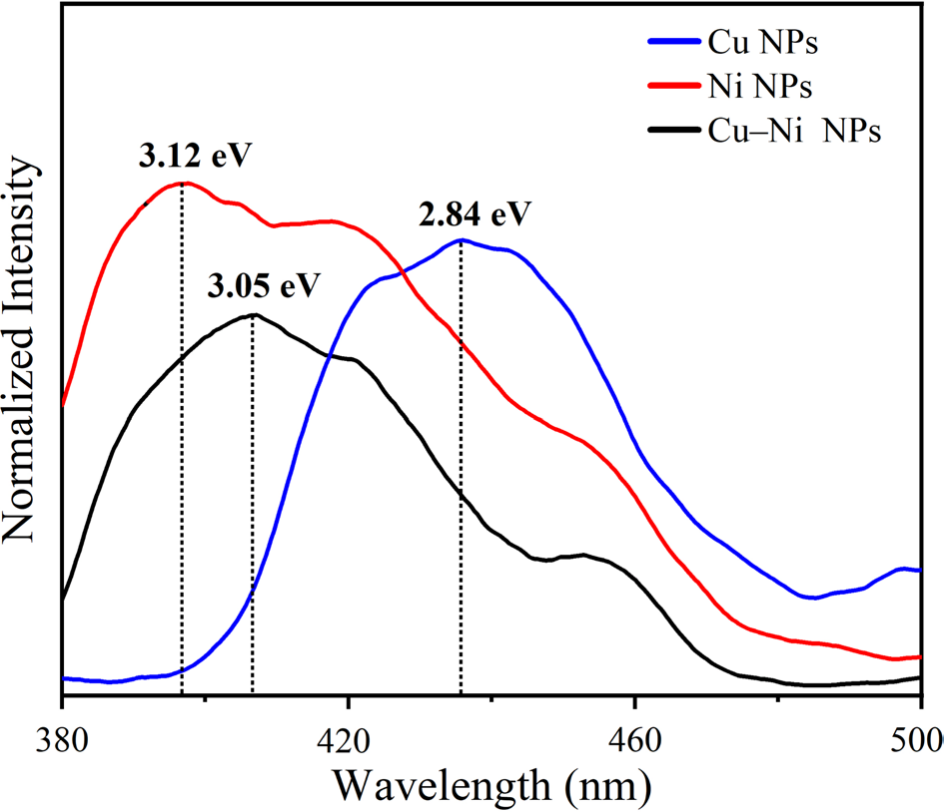
PL emission spectra of as-prepared nanoparticle colloids

MNPs are also capable of photoluminescence (PL) emission in visible region. It is due to the radiative recombination of excited electrons in the sp band (CB) with the holes in the d band (VB), after an energy loss because of the electron–phonon and hole-phonon scattering process [52, 53]. Figure 7 represents the room temperature PL emission spectra of as-prepared samples under the excitation wavelength of 350 nm in the range of 380–500 nm. The values of band gaps measured from PL spectra are not necessarily the same as those obtained from absorption analysis (Tauc plots), representing that different mechanisms are involved in the emission and absorption transitions. Emitted energy from radiative transition is equal to the energy difference between the ground and exited levels. However, the emitted energy is often lower than the absorbed energy, due to the initial relaxation and non-radiative transitions of the excited electrons in the sp band. All colloidal solutions exhibited intensive PL emission peaks around 400 nm. The photoemission peaks at the blue region indicate that synthesized NPs are blue luminescent in nature. Table 1 compares the average particle size, crystallite size, energy band gap, and SPR and PL peak positions of PLAL-asserted prepared NPs with other findings reported in the literature.

## Conclusion

Highly crystalline and homogeneously dispersed spherical copper, nickel, and bimetallic nanoparticles were prepared in DI water using a simple, precise, and eco-friendly PLAL method. The production of nanocrystals with optimal morphology indicated the effectiveness of PLAL method and DI water as a growth medium. The structural, optical, and morphological properties of produced NPs were analyzed using different techniques at room temperature. Cu–Ni bimetallic NPs with a Ni-rich core and Cu-rich shell were successfully synthesized using PLAL technique. The size distribution of Cu NPs, Ni NPs, and Cu–Ni NPs was uniform with the corresponding mean diameters of 7.2 nm, 10.2 nm, and 9.5 nm. The optical band gap energies of prepared NPs were estimated from the UV absorption edge of the UV–Vis spectra and further verified by the PL spectral analysis. A well-defined LSPR peaks for Cu and Cu–Ni colloidal solutions were recorded at 645 nm and 596 nm wavelengths, indicating their usefulness for plasmonic applications. A comparative evaluation of some optical and morphological properties of these PLAL-assisted synthesized Cu–Ni NPs with the same bimetallic NPs produced by other methods showed that the proposed NPs with smaller size, spherical shape, high crystallinity, and enhanced optical properties could be advantageous for various practical applications.

## Supplementary Information

Supplementary file1 (DOCX 5164 KB)

## Data Availability

Data is provided within the manuscript or supplementary information files.
