# Supplementary material for: Facile synthesis of copper, nickel and their bimetallic nanoparticles: optical and structural characterization
Source: Discov Nano. 2025 Feb 11;20(1):28. doi: 10.1186/s11671-025-04197-8 (PMC11813849; doi:10.1186/s11671-025-04197-8)
Supplement: Supplementary file 1 — Supplementary file1 (DOCX 5164 KB) [file 11671_2025_4197_MOESM1_ESM.docx]

**Supporting Information**

**Facile synthesis of copper, nickel and their alloy nanocrystals: Optical and structural characterization**

Abdul Waheed Aman^1,2*^, Ganesan Krishnan^2^, Mohammad Abdullah Sadiqi^1^, Mahmood Alhajj^2^, Nurul Hidayat^3,4^

^1^Department of Physics, Faculty of Education, Helmand University, Lashkar Gah 3901, Helmand, Afghanistan

^2^Department of Physics & Laser Center, Faculty of Science, Universiti Teknologi Malaysia, 81310, Johor, Malaysia

^3^Department of Physics, Faculty of Mathematics and Natural Sciences, Universitas Negeri Malang, Jl. Semarang 5, Malang 65145, Indonesia

^4^Center of Science and Engineering, Universitas Negeri Malang, Jl. Semarang 5, Malang 65145, Indonesia

**Elemental Composition Analysis**

b

a


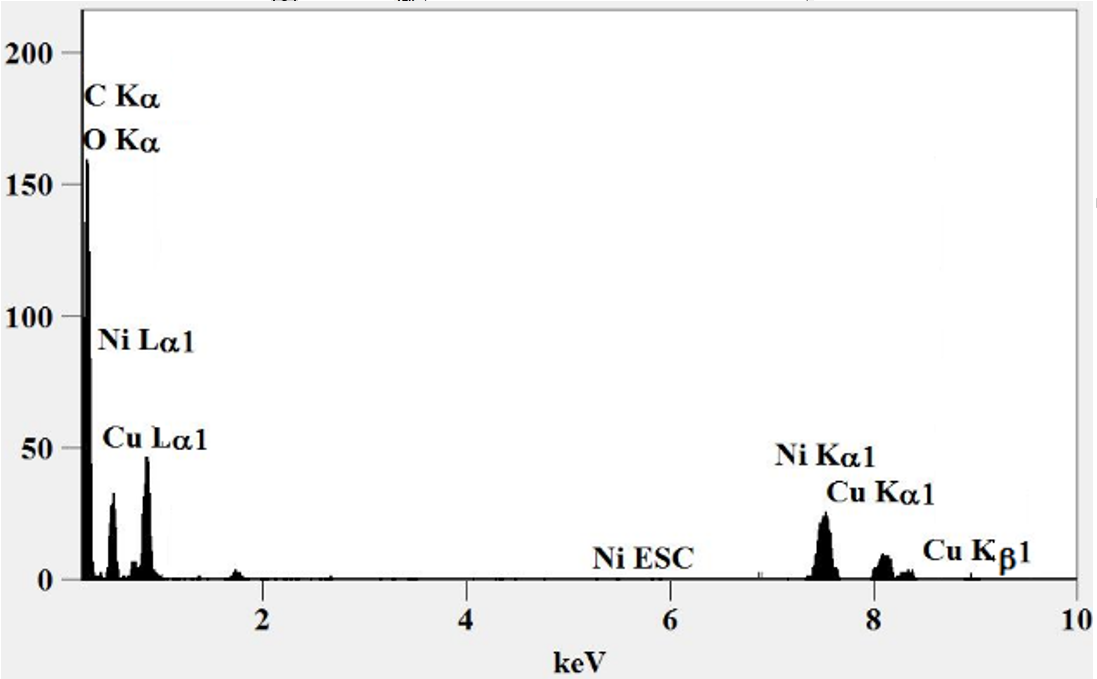

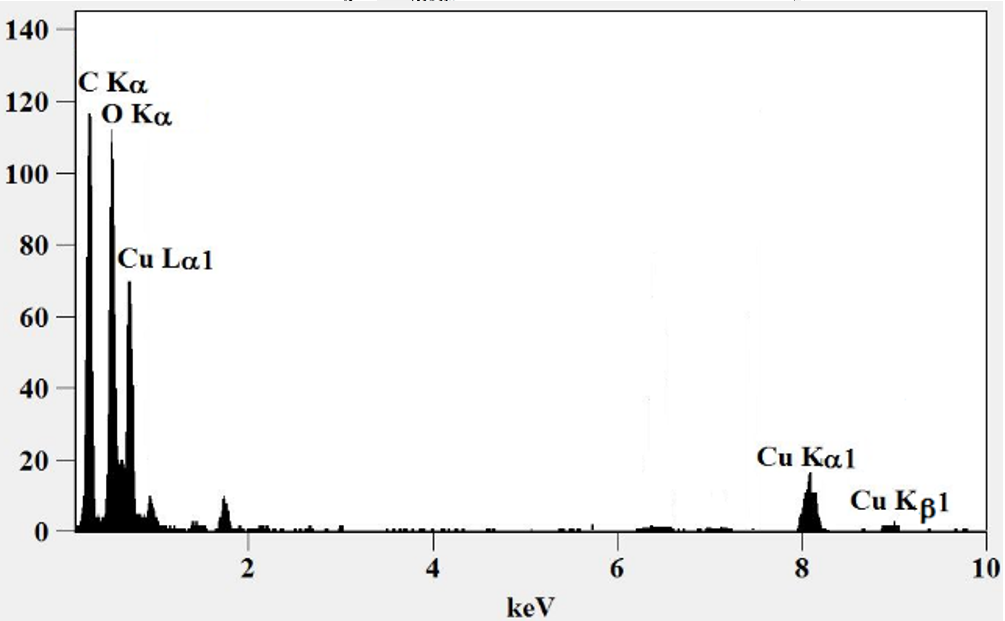


**Fig. S1** Energy-dispersive X-ray (EDX) results of synthesized nanoparticles: **a** Cu NPs, **b** Ni NPs, and **c** Cu–Ni NPs

c


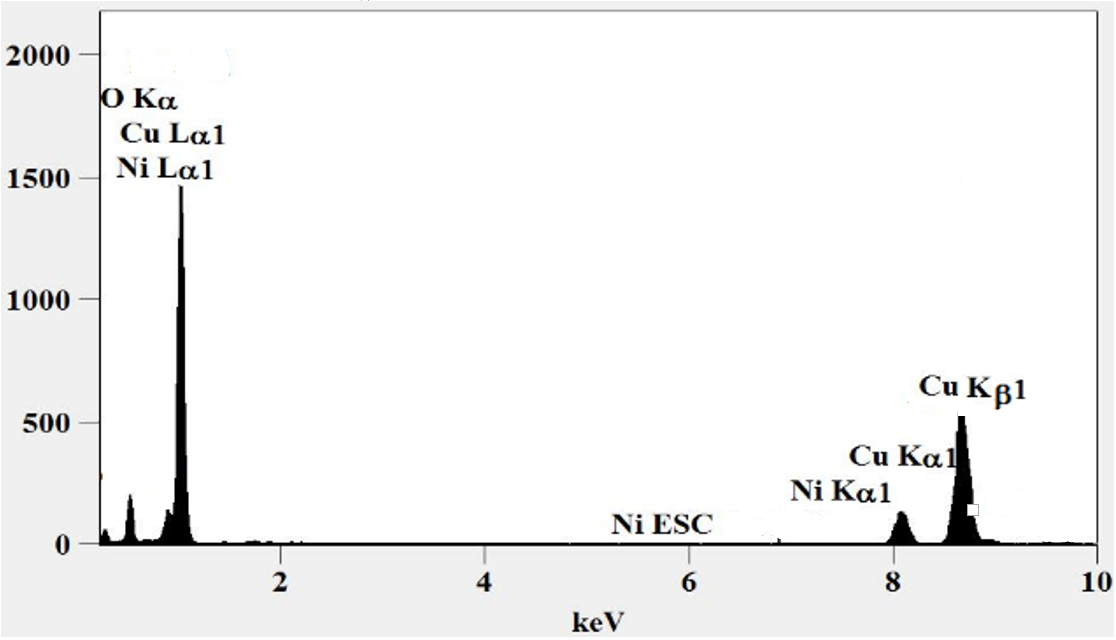


**Temporal evolution of LSPR peak**

b

a


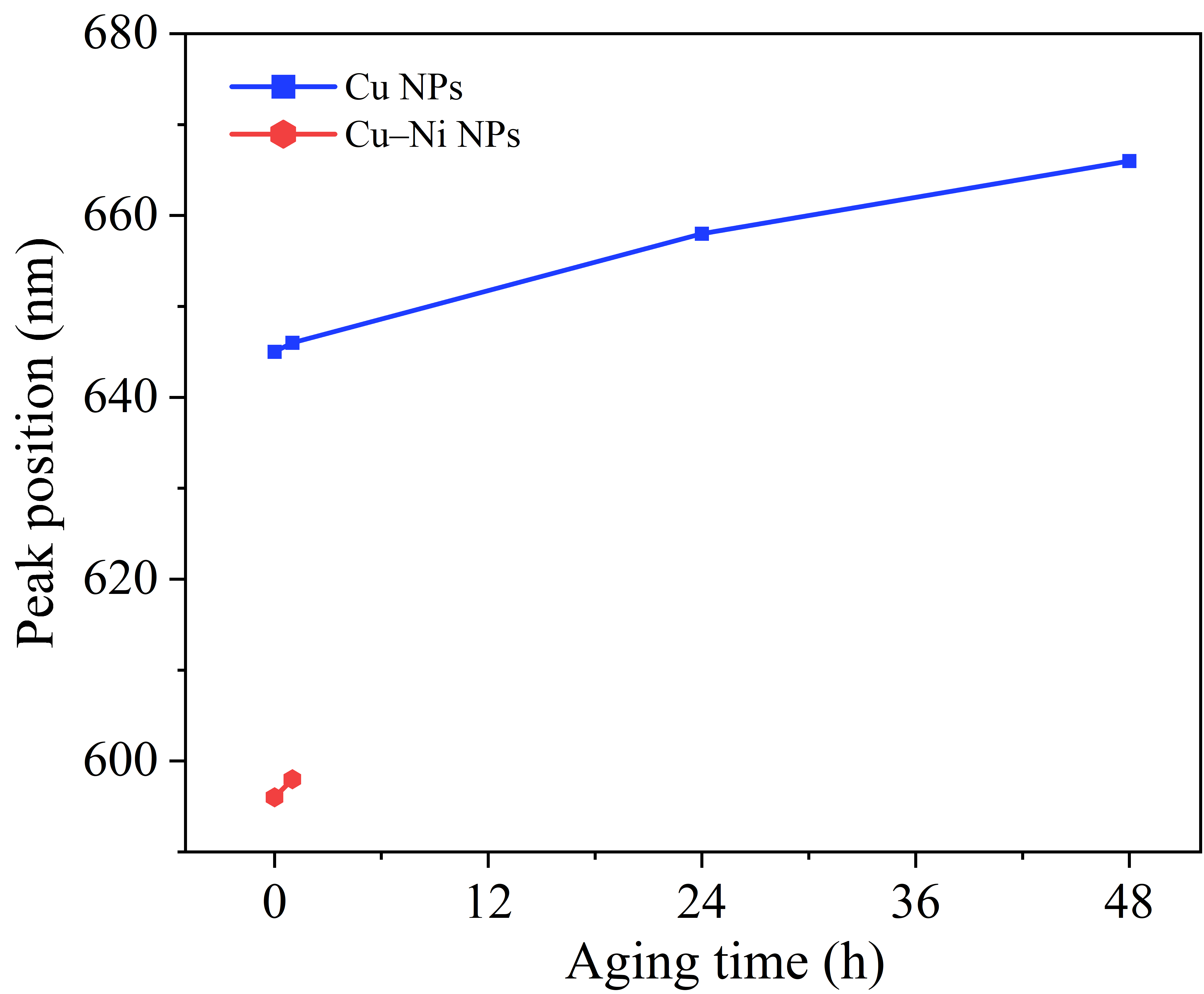

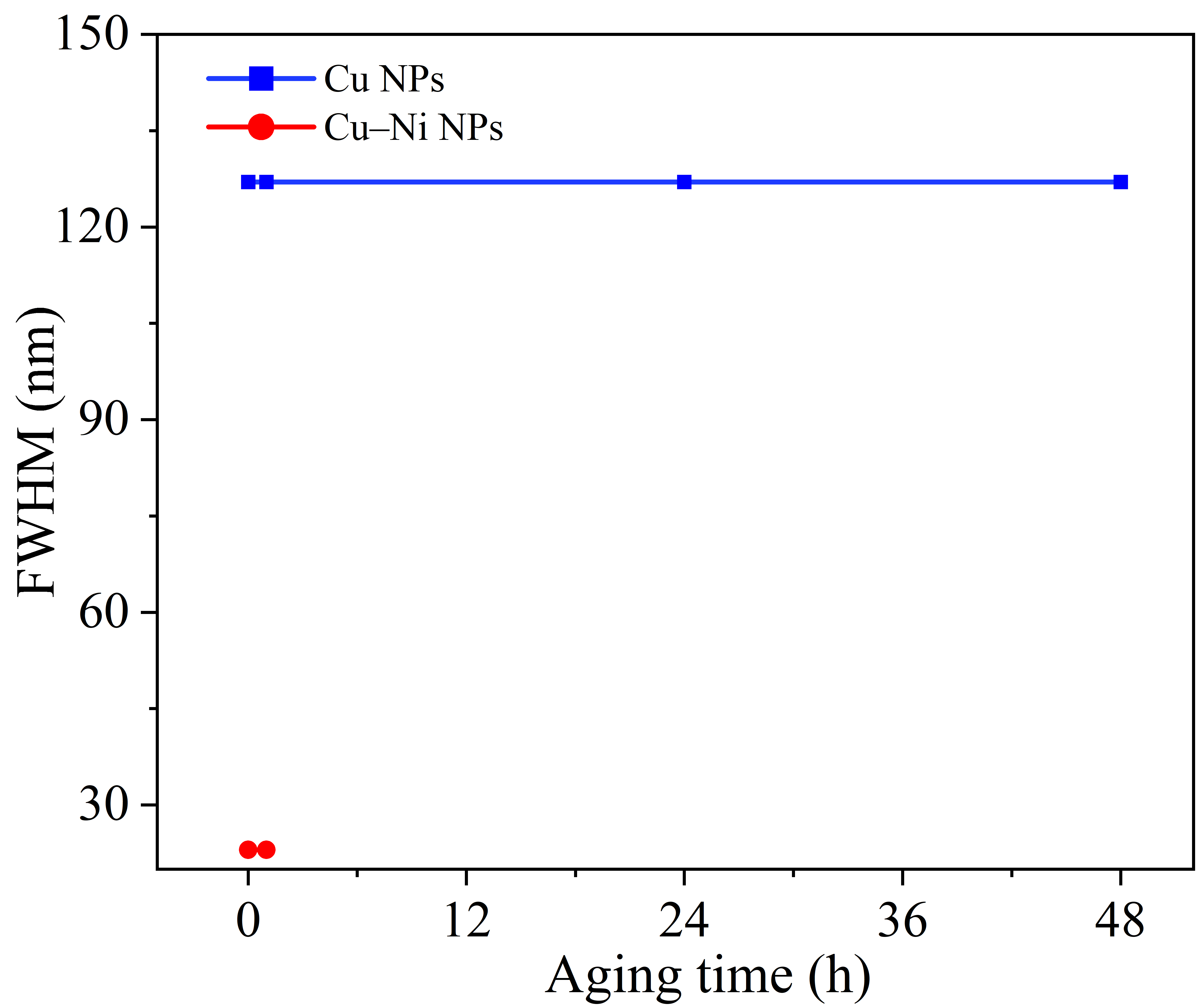


c


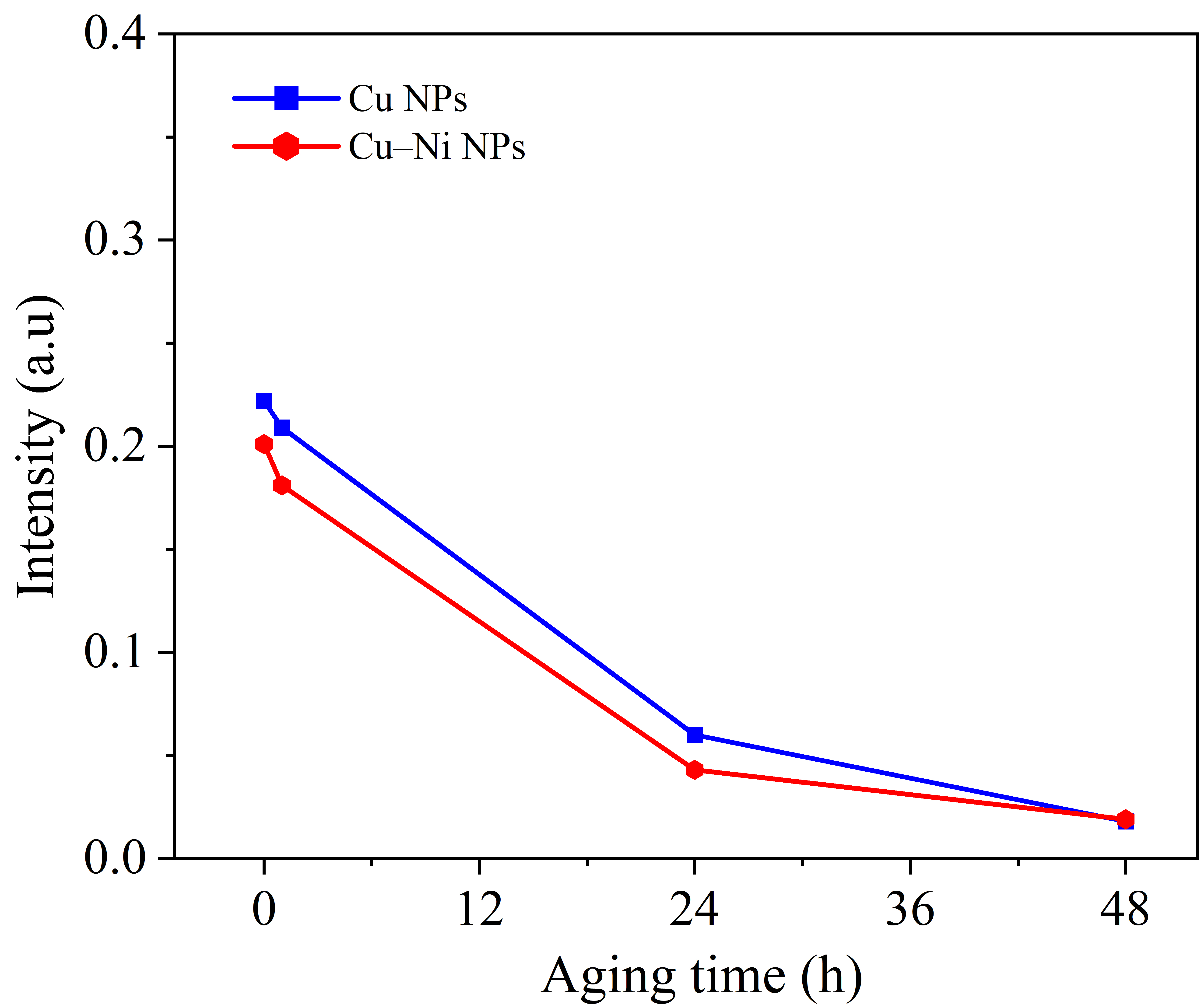


**Fig. S2** The LSPR peak **a** position, **b** FWHM, and **c** intensity as a function of aging time of Cu NPs Cu–Ni NPs

**Lattice spacing measurement**

**Fig. S3** Inverse fast Fourier transform (IFFT) patterns and IFFT profile of measured lattice fringes of corresponding HR-TEM images: **a** Cu NPs, **b** Ni NPs, and **c** and **d** Cu–Ni NPs


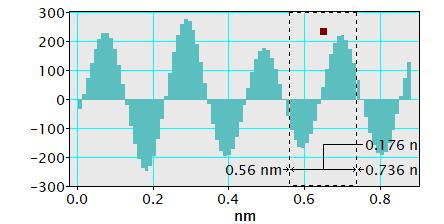

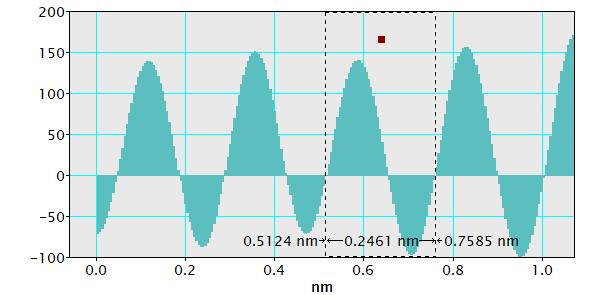

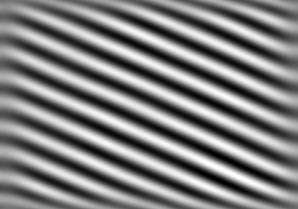

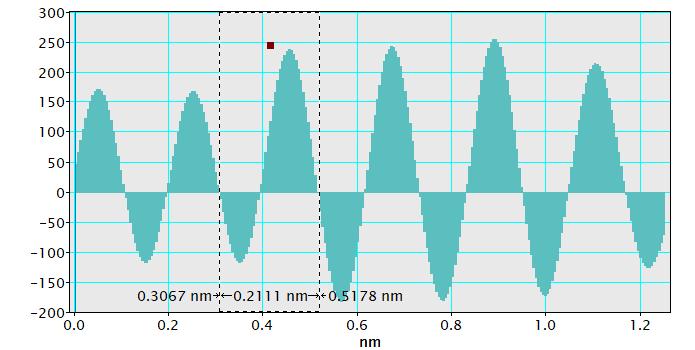

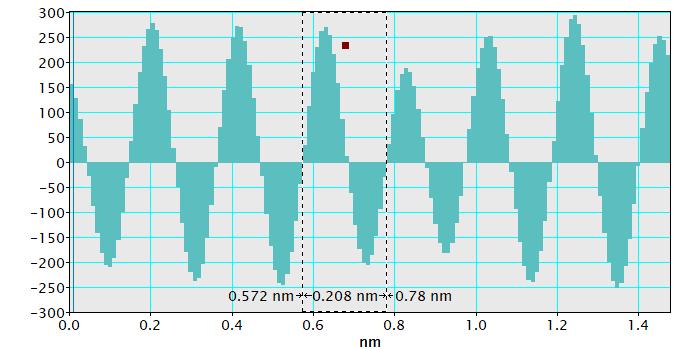

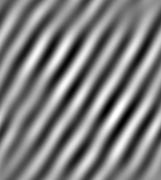

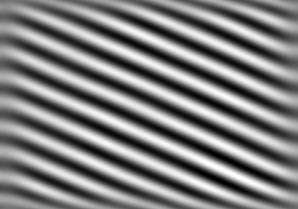

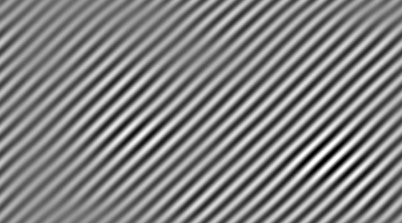


a

b

c

d
